# Identifying systematic reviews of the adverse effects of health care interventions

**DOI:** 10.1186/1471-2288-6-22

**Published:** 2006-05-08

**Authors:** Su Golder, Heather M McIntosh, Yoon Loke

**Affiliations:** 1 Centre for Reviews and Dissemination (CRD), University of York, York, YO10 5DD, UK; 2 NHS Quality Improvement Scotland, Delta House, 50 West Nile St, Glasgow, G1 2NP, UK; 3 University of East Anglia Norwich NR4 7TJ, UK

## Abstract

**Background:**

In order to carry out a methodological research survey of systematic reviews of adverse effects we needed to retrieve a sample of systematic reviews in which the primary outcome is an adverse effect or effects.

**Methods:**

We carried out searches of the Database of Abstracts of Reviews of Effects (DARE) and the Cochrane Database of Systematic Reviews (CDSR) for systematic reviews of adverse effects published between 1994 to 2005. The search strategies used a combination of text words in the title and abstract, Medical Subject Headings (MeSH) and subheadings/qualifiers. In addition, DARE records in progress were hand searched. No language restrictions were placed on any of the searches. The performance, in terms of sensitivity and precision, of the search strategies and their combinations were tested in DARE and CDSR.

**Results:**

In total 3635 records were screened of which 257 met our inclusion criteria. The precision of the searches in CDSR was low (0% to 3%), and no one search strategy could retrieve all the relevant records in either DARE or CDSR. Hand searching the records from DARE and CDSR not retrieved by our searches indicated that we had missed relevant systematic reviews in both DARE and CDSR. The sensitivities of many of the search combinations were comparable to those found when searching for primary studies in which adverse effects are secondary outcomes.

**Conclusion:**

Searching major databases of systematic reviews, for systematic reviews of adverse effects, proved more difficult than anticipated due to a lack of standard terminology used by the authors, inadequate indexing and the variations in the search interfaces of the databases. At present hand searching all records in DARE and CDSR seems to be the only way to ensure retrieval of all systematic reviews of adverse effects in these databases.

## Background

Balanced decision making in health care requires evidence on the potential adverse effects of interventions as well as their beneficial effects. Although well-conducted systematic reviews of adverse effects are important sources of evidence such reviews are relatively rare in the literature [1] and it is not clear whether the process of identifying relevant reviews may resemble the proverbial "looking for a needle in the haystack". Indeed, poor indexing and inconsistent terminology have hampered efforts to identify studies that report original data on adverse effects [2-5]. These primary studies often do not consider adverse effects as the main outcome and, therefore, may not contain this information in their title or abstract or be indexed with terms for adverse effects.

It should be easier to identify systematic reviews that were conducted with the express purpose of evaluating adverse effects. We might expect that study retrieval would be facilitated by some mention of adverse effects in the title, abstract or indexing terms. As part of a wider study of methods used in systematic reviews of adverse effects we decided to assess whether we could identify systematic reviews of adverse effects quickly and easily in two major databases of systematic reviews.

## Methods

We searched for systematic reviews of adverse effects using the Database of Abstracts of Reviews of Effects (DARE) and the Cochrane Database of Systematic Reviews (CDSR). These databases were chosen because they are major collections of systematic reviews. No additional sources were searched as DARE is compiled through rigorous monthly searches of bibliographic databases (including MEDLINE and EMBASE) as well as hand searching key journals, grey literature, and regular searches of the web [6]. No language restrictions were placed on the searches and the searches aimed to retrieve systematic reviews published from 1994 onwards.

### Database of Abstracts of Reviews of Effects (DARE)

**Figure 1:**
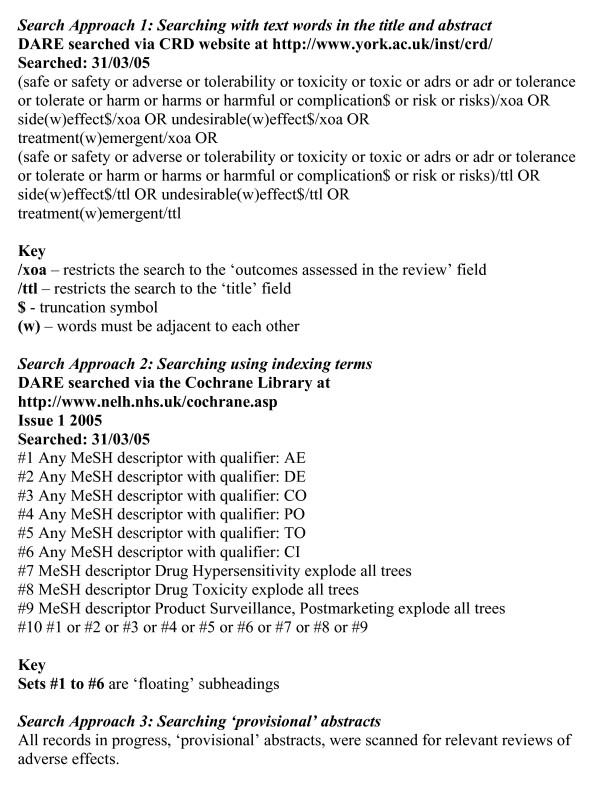
Search strategies for retrieving systematic reviews of adverse effects from DARE.

Three approaches were used to identify records in DARE (figure 1). Firstly, text word searches for synonyms of 'adverse effects' and related terms were carried out in the record title and abstract. These terms were selected from previous research [4]. Each DARE abstract contains a summary of a systematic review including a critical commentary. It was found that searching the full abstracts of DARE records would have identified many irrelevant records as these abstracts contain phrases such as; 'no information on the incidence of adverse reactions are included', 'it would have been appropriate to include mention of adverse events' or 'the adverse effects of the treatment were not assessed in the review'. The searches of the DARE abstracts were, therefore, restricted to the 'outcomes assessed in the review' field as the primary outcome of a review is described in this field [7].

The second approach was to use Medical Subject Headings (MeSH), such as DRUG TOXICITY, and subheadings/qualifiers unattached to any indexing terms ('floating' subheadings), such as 'adverse effects'. This was an essential part of the search strategy as many systematic reviews examine specific adverse effects, such as, headaches, so would not necessarily be identified by text words of synonyms of 'adverse effects' and searching for each named potential adverse effect individually would be impractical. Previous research has also indicated the usefulness of searching with 'floating' adverse effect subheadings [2-4].

Finally, DARE records in the process of being written do not yet have an 'outcomes assessed in the review' field. The titles of all of these 'provisional' records were, therefore, hand searched by the researchers to identify additional relevant reviews.

To enable all three approaches to be executed, three searches of DARE were conducted, two via the Centre for Reviews and Dissemination (CRD) website and one via The Cochrane Library website (figure 1). The text word search was conducted using the CRD website because this interface allows searches to be limited to sections of the structured abstracts, such as, the 'outcomes assessed in the review' field, whereas The Cochrane Library interface does not. The provisional abstracts were scanned via the CRD website as this contains the most up to date set of provisional DARE records. Another search was conducted using The Cochrane Library because its interface allows searches of 'floating' subheadings to be conducted whereas the CRD website does not.

### The Cochrane Database of Systematic Reviews (CDSR)

**Figure 2:**
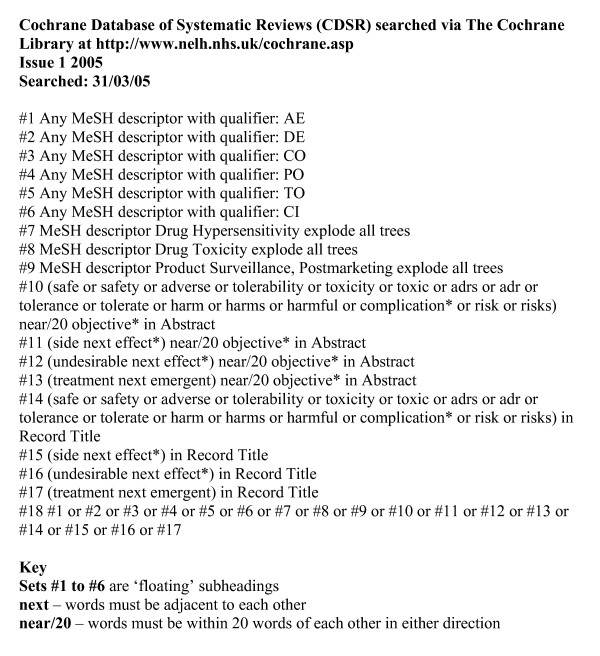
Search strategies for retrieving systematic reviews of adverse effects from CDSR.

Searches for Cochrane Reviews were conducted in the web version of The Cochrane Library (Issue 1: 2005) (figure 2). These searches used text words in the title and abstract, MeSH terms, and 'floating' subheadings. As with DARE records many irrelevant records would have been retrieved if the full CDSR structured abstracts had been searched. The 'objectives' section of a CDSR abstract outlines the primary outcome of a review [8]. As searches in CDSR cannot be limited to sections of the abstract text words were searched for in the abstract using the proximity operator to limit to within 20 words of the term 'objectives' (figure 2).

### Inclusion criteria

The results from all four searches were then entered into an Endnote Library and duplicate records were removed. Two researchers independently screened the titles and abstracts and selected records for inclusion in the study. A review was included if the primary outcome was an adverse effect or effects, that were known to be, or suspected of being, associated with the intervention. This was regardless of whether the review indicated that the intervention increased or reduced the outcome.

It was suspected that relevant reviews had been missed by the searches so those records not retrieved by the search strategies in CDSR (n = 887) and DARE (n = 2646) were also scanned for relevant systematic reviews. All relevant records identified then formed our gold standard (GS) set of records.

### Assessing the performance of the search strategies

Once we had established our gold standard set of records we were able to test the performance of individual approaches in retrieving the gold standard records. The search terms used to identify the systematic reviews were assessed for their usefulness in retrieving relevant records by measuring their sensitivity and precision. Sensitivity is a measure of the search's ability to identify relevant papers, and a high value is important for searches for systematic reviews. Precision, on the other hand, is a measure of the proportion of relevant records identified by a search strategy expressed as a percentage of all articles (relevant and irrelevant) identified by that strategy. Highly sensitive strategies tend to have low levels of precision. Sensitivity and precision for each database were calculated as follows;

Sensitivity=number of GS records retrievednumber of GS records indexedin the database under investigation×100
 MathType@MTEF@5@5@+=feaafiart1ev1aaatCvAUfKttLearuWrP9MDH5MBPbIqV92AaeXatLxBI9gBaebbnrfifHhDYfgasaacH8akY=wiFfYdH8Gipec8Eeeu0xXdbba9frFj0=OqFfea0dXdd9vqai=hGuQ8kuc9pgc9s8qqaq=dirpe0xb9q8qiLsFr0=vr0=vr0dc8meaabaqaciaacaGaaeqabaqabeGadaaakeaacqqGtbWucqqGLbqzcqqGUbGBcqqGZbWCcqqGPbqAcqqG0baDcqqGPbqAcqqG2bGDcqqGPbqAcqqG0baDcqqG5bqEcqGH9aqpdaWcaaqaaiabb6gaUjabbwha1jabb2gaTjabbkgaIjabbwgaLjabbkhaYjabbccaGiabb+gaVjabbAgaMjabbccaGiabbEeahjabbofatjabbccaGiabbkhaYjabbwgaLjabbogaJjabb+gaVjabbkhaYjabbsgaKjabbohaZjabbccaGiabbkhaYjabbwgaLjabbsha0jabbkhaYjabbMgaPjabbwgaLjabbAha2jabbwgaLjabbsgaKbabaeqabaGaeeOBa4MaeeyDauNaeeyBa0MaeeOyaiMaeeyzauMaeeOCaiNaeeiiaaIaee4Ba8MaeeOzayMaeeiiaaIaee4raCKaee4uamLaeeiiaaIaeeOCaiNaeeyzauMaee4yamMaee4Ba8MaeeOCaiNaeeizaqMaee4CamNaeeiiaaIaeeyAaKMaeeOBa4MaeeizaqMaeeyzauMaeeiEaGNaeeyzauMaeeizaqgabaGaeeyAaKMaeeOBa4MaeeiiaaIaeeiDaqNaeeiAaGMaeeyzauMaeeiiaaIaeeizaqMaeeyyaeMaeeiDaqNaeeyyaeMaeeOyaiMaeeyyaeMaee4CamNaeeyzauMaeeiiaaIaeeyDauNaeeOBa4MaeeizaqMaeeyzauMaeeOCaiNaeeiiaaIaeeyAaKMaeeOBa4MaeeODayNaeeyzauMaee4CamNaeeiDaqNaeeyAaKMaee4zaCMaeeyyaeMaeeiDaqNaeeyAaKMaee4Ba8MaeeOBa4gaaaGaey41aqRaeGymaeJaeGimaaJaeGimaadaaa@B8B6@

Precision=number of GS records retrievedtotal number of records retrieved×100
 MathType@MTEF@5@5@+=feaafiart1ev1aaatCvAUfKttLearuWrP9MDH5MBPbIqV92AaeXatLxBI9gBaebbnrfifHhDYfgasaacH8akY=wiFfYdH8Gipec8Eeeu0xXdbba9frFj0=OqFfea0dXdd9vqai=hGuQ8kuc9pgc9s8qqaq=dirpe0xb9q8qiLsFr0=vr0=vr0dc8meaabaqaciaacaGaaeqabaqabeGadaaakeaacqqGqbaucqqGYbGCcqqGLbqzcqqGJbWycqqGPbqAcqqGZbWCcqqGPbqAcqqGVbWBcqqGUbGBcqGH9aqpdaWcaaqaaiabb6gaUjabbwha1jabb2gaTjabbkgaIjabbwgaLjabbkhaYjabbccaGiabb+gaVjabbAgaMjabbccaGiabbEeahjabbofatjabbccaGiabbkhaYjabbwgaLjabbogaJjabb+gaVjabbkhaYjabbsgaKjabbohaZjabbccaGiabbkhaYjabbwgaLjabbsha0jabbkhaYjabbMgaPjabbwgaLjabbAha2jabbwgaLjabbsgaKbqaaiabbsha0jabb+gaVjabbsha0jabbggaHjabbYgaSjabbccaGiabb6gaUjabbwha1jabb2gaTjabbkgaIjabbwgaLjabbkhaYjabbccaGiabb+gaVjabbAgaMjabbccaGiabbkhaYjabbwgaLjabbogaJjabb+gaVjabbkhaYjabbsgaKjabbohaZjabbccaGiabbkhaYjabbwgaLjabbsha0jabbkhaYjabbMgaPjabbwgaLjabbAha2jabbwgaLjabbsgaKbaacqGHxdaTcqaIXaqmcqaIWaamcqaIWaamaaa@8FD9@

## Results

**Figure 3:**
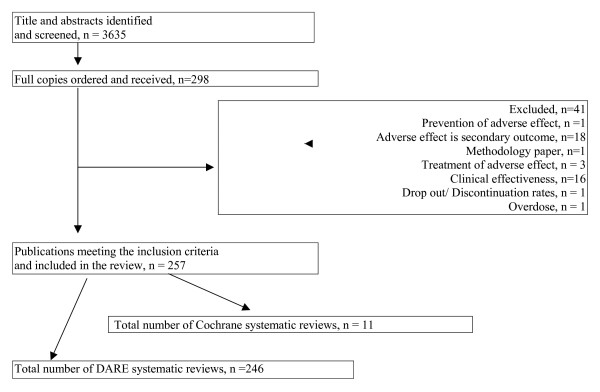
Summary of systematic review identification, retrieval and inclusion/exclusion.

In total 4262 records were retrieved from CDSR and DARE, of which 3635 were unique records. From the 3635 titles and abstracts screened, 298 full reports were retrieved and 256 reviews (257 publications) met our inclusion criteria. Of the 257 publications, 246 had DARE abstracts and 11 were Cochrane Reviews (figure 3).

The hand search of the records in CDSR and DARE not retrieved by our search strategies identified 13 additional records (10 from DARE and 3 from CDSR) which met our inclusion criteria. In total, therefore, 270 systematic reviews of adverse effects were identified; 256 from DARE and 14 from CDSR.

The relevant records not retrieved by our search strategies were sifted for any potentially relevant generic adverse effect search terms. Only 2 of the 13 contained potentially useful terms. Both contained the MeSH indexing term RISK FACTORS and one had the term 'hazards' in the title. These search terms, in addition to the terms used in our search strategies, were tested to identify the most sensitive search strategy possible.

The sensitivity and precision of the different search approaches are presented in table 1. Searching using 'floating' subheadings provided the highest sensitivity in both DARE (85%) and CDSR (64%) and the precision of all the search approaches was much higher in DARE (16% to 71%) than in CDSR (0% to 3%).

**Table 1:** Sensitivity and precision of searches in DARE and CDSR

	No of Papers Retrieved	No of Relevant Papers	Sensitivity (%)	Precision (%)
**DARE (Quasi Gold Standard = 256)**				
'floating' subheadings: 'adverse effects' OR 'drug effects' OR 'complications' OR 'poisoning' OR 'toxicity' OR 'chemically induced'	1386	217	85%	16%
'floating' subheadings: 'adverse effects' OR 'drug effects' OR 'complications' OR 'poisoning', 'toxicity' OR 'chemically induced' OR Exp DRUG HYPERSENSITIVITY OR Exp DRUG TOXICITY OR Exp PRODUCT SURVEILLANCE, POSTMARKETING	1386	217	85%	16%
synonyms of 'adverse effects' in the 'title' OR 'outcomes assessed in the review' field	873	160	63%	18%
synonyms of 'adverse effects' in the title	462	132	52%	29%
synonyms of 'adverse effects' in 'outcomes assessed in the review' field	659	116	45%	18%
exp DRUG HYPERSENSITIVITY OR exp DRUG TOXICITY OR exp PRODUCT SURVEILLANCE, POSTMARKETING	14	10	4%	71%
**CDSR (Quasi Gold Standard = 14)**				
'floating' subheadings: 'adverse effects' OR 'drug effects' OR 'complications' OR 'poisoning', 'toxicity' OR 'chemically induced'	416	9	64%	3%
synonyms of 'adverse effects' near/20 objectives in the 'abstract' field	1049	5	36%	0.5%
synonyms of 'adverse effects' in the 'title' field	64	2	14%	3%
exp DRUG HYPERSENSITIVITY OR exp DRUG TOXICITY OR exp PRODUCT SURVEILLANCE, POSTMARKETING	2	0	0%	0%

### Single search terms with the highest precision

All the single search terms in CDSR yielded very low precision (0 to 3%). In DARE, however, some terms did provide high precision (table 2). The single terms with the highest precision were the MeSH terms, Exp PRODUCT SURVELLIENCE, POSTMARKETING (73%), Exp DRUG HYPERSENSITIVITY (67%) and Exp DRUG TOXICITY (67%). However, the sensitivity of searching with each of these terms was very low (1–3%) (table 2). Searches using all the chosen synonyms of 'adverse effects' in the title had a reasonable precision of 29% (table 1). However, some individual single terms yielded higher precision. For example, searching with the term 'adverse' in the title gave 65% precision, 'complication$' 50% and 'side effect$' 43%.

**Table 2:** Single search terms with highest precision in DARE (Quasi Gold Standard = 256)

Search Term	Field Searched	No of Papers Retrieved	No of Relevant Papers	Precision (%)	Sensitivity (%)
exp DRUG HYPERSENSITIVITY	indexing	11	8	73%	3%
exp DRUG TOXICITY	indexing	3	2	67%	1%
exp PRODUCT SURVEILLANCE, POSTMARKETING	indexing	3	2	67%	1%
adverse	title	40	26	65%	10%
complication$	title	28	14	50%	5%
side effect$	title	7	3	43%	1%
'toxicity' (floating subheading)	indexing	7	3	43%	1%
'chemically-induced' (floating subheading)	indexing	201	85	42%	33%
adverse	outcomes assessed	78	32	41%	13%
risk	title	140	55	39%	21%
side effect$	outcomes assessed	3	1	33%	0%
risk	outcomes assessed	168	54	32%	21%
safety	title	96	29	30%	11%
complication$	outcomes assessed	46	13	28%	5%
risks	outcomes assessed	39	10	26%	4%

### Single search terms with the highest sensitivity

The most sensitive search strategy used 'floating' subheadings (table 1). 'Floating' all the subheadings retrieved 85% of the DARE records and 64% of the Cochrane Reviews. The most sensitive 'floating' subheadings in DARE were 'adverse-effects' at 77%, followed by 'chemically-induced' (33%), 'drug-effects' (16%), and 'complications' (14%) (table 3). In CDSR 'adverse-effects' was again the most sensitive 'floating' subheading (64%), followed by 'chemically-induced' (29%), and 'drug-effects' (29%).

**Table 3:** Single search terms with the highest sensitivity in DARE (Quasi Gold Standard = 256)

Search Term	Field Searched	No of Papers Retrieved	No of Relevant Papers	Sensitivity (%)	Precision (%)
'adverse-effects' ('floating' subheading)	indexing	898	198	77%	22%
'chemically-induced' ('floating' subheading)	indexing	201	85	33%	42%
RISK FACTORS	indexing	336	69	27%	21%
risk	title	140	55	21%	39%
risk	outcomes Assessed	168	54	21%	32%
'drug-effects' (floating subheading)	indexing	273	42	16%	15%
'complications' (floating subheading)	indexing	550	36	14%	7%
adverse	outcomes assessed	78	32	13%	41%
safety	title	96	29	11%	30%
adverse	title	40	26	10%	65%

### Most sensitive search strategies

**Figure 4:**
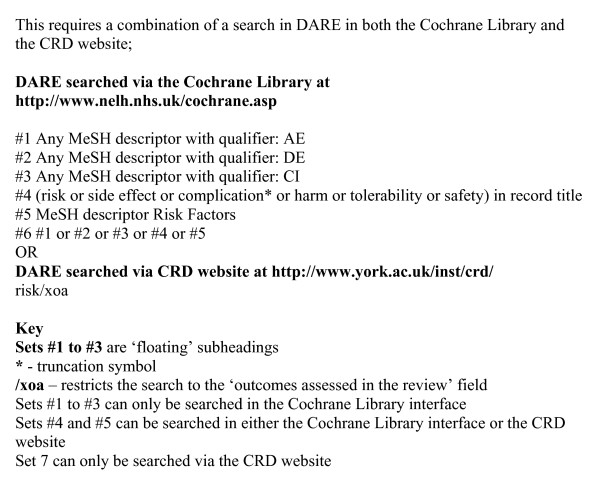
Most sensitive search strategy to retrieve adverse effects in DARE.

The most sensitive search strategy in DARE, with the terms tested here, used a combination of text words in the title and abstract, a MeSH term and 'floating' subheadings (see figure 4). This strategy retrieved 1,507 records of which 241 were deemed relevant, yielding a sensitivity of 94% and precision of 16%.

In CDSR the most sensitive search strategy used the 'floating' subheading 'adverse effects' combined with searching for 'adverse near/20 objectives' in the abstract. This strategy retrieved 79% (11/14) of the relevant records. However, the precision of this search was low at 3% (11/338).

## Discussion

This research highlights the advantages and disadvantages of searching databases through the CRD website and The Cochrane Library website. The CRD website offered the most current version of DARE and allowed searches to be limited to sections of the abstract, whereas The Cochrane Library version of DARE allowed searching using 'floating' subheadings. Even when conducting consecutive searches on DARE and CDSR in two different interfaces it is difficult to retrieve all systematic reviews of adverse effects on these databases. A sensitive search using text words in the title and abstract, indexing terms and 'floating' subheadings was unable to retrieve all the records of interest. An assessment of the missed systematic reviews indicated that most of these records could not have been retrieved without searching for specific adverse effects. Although adding these terms to our search strategy would have increased the sensitivity of the searches, adding the MeSH term RISK FACTORS, in particular, would have decreased the precision.

Research has indicated that primary studies of adverse effects are difficult to locate [2-5]. This has been attributed to poor reporting, inconsistent terminology and inadequate indexing. In primary studies adverse effects are often not the main outcome of the study and are described secondary to clinical effectiveness. In this case study we sought systematic reviews in which the primary outcome was an adverse effect or effects. It was anticipated that such studies would be easier to retrieve because adverse effects would more likely be contained in their title and abstract and thus their indexing. This was tested by comparing the sensitivities and precision of our searches to those reported in earlier research on primary studies.

Interestingly our searches shared similar sensitivities to those reported by Badgett et al's [2] and Golder et al's [4] when searching with subheadings and indexing terms (table 4). The single most sensitive term in all three studies was the 'floating' subheading 'adverse effects' (table 4). It is difficult to compare the sensitivities of searching in the abstract as in this study the searches were limited to particular sections of the structured abstracts in DARE and CDSR. Searching the title for synonyms of 'adverse effects' and related terms showed a higher sensitivity in DARE in this study than in Badgett et al's [2] and Golder et al's [4]. This may reflect the fact that the relevant studies here contained an adverse effect or effects as their primary outcome. The lower sensitivity we experienced in CDSR, however, may reflect the prescribed format of Cochrane Review titles which focus on the intervention and condition [8].

**Table 4:** Comparison of search sensitivities in three case studies

Search Terms	Sensitivity in DARE in this case study (GS = 256)	Sensitivity in CDSR in this case study (GS = 14)	Sensitivity in MEDLINE in Badgett et al's study [2] (QGS = 323)	Sensitivity in MEDLINE in Golder et al's study [4] (QGS = 67)	Sensitivity in EMBASE in Golder et al's study [4] (QGS = 72)
'adverse effects' (floating subheading)	77%	64%	86%	79%	79%[1]
'complications' (floating subheading)	14%	0%	12%	6%	5%[1]
'poisoning' (floating subheading)	0.4%	0%	10%	0%	n/a
'chemically induced' (floating subheading)	33%	29%	Not tested	28%	n/a
'toxicity' (floating subheading)	1%	0%	Not tested	0%	3%[1]
'drug effects' (floating subheading)	16%	29%	Not tested	33%	n/a
'adverse effects' OR 'complications' OR 'poisoning' (floating subheadings)	79%	57%	95%	82%	81%
'adverse effects OR 'complications' OR 'poisoning' OR 'drug effects' OR 'toxicity' OR 'chemically induced' (floating subheadings)	85%	64%	Not tested	88%	81%[1]
'adverse effects' OR 'drug effects' OR 'complications' OR 'poisoning' OR 'toxicity' OR 'chemically induced' (all floating subheadings) OR exp DRUG HYPERSENSITIVITY OR exp DRUG TOXICITY OR exp PRODUCT SURVEILLANCE, POSTMARKETING	85%	64%	Not tested	88%	81%[1]
exp DRUG HYPERSENSITIVITY OR exp DRUG TOXICITY OR exp PRODUCT SURVEILLANCE, POSTMARKETING	4%	0%	Not tested	10%	7%
text word 'adverse' in the title or abstract	16%[2]	29%[2]	10%	54%	50%
synonyms of 'adverse effects' in the title	52%	7%	Not tested	18%	19%
synonyms of 'adverse effects' in the abstract	45%[2]	36%[2]	Not tested	87%	89%
synonyms of 'adverse effects' in the title Or abstract	63%[2]	36%[2]	Not tested	75%	75%

[1] the subheadings 'adverse drug reaction', drug toxicity' and ' complication' were used in EMBASE as nearest equivalent to 'adverse effects', 'toxicity' and 'complications'. [2] searches in DARE and CDSR were limited to sections of the structured abstracts.

Derry et al [3] found that of a sample of randomised controlled trials (RCTs) reporting adverse effects, only 77% (82/107) could be identified by adverse effects indexing terms or text words in the title or abstract. In our study, 79% (11/14) of the Cochrane Reviews and 91% (233/256) of the DARE reviews could be identified by adverse effects indexing terms or text words in the title or abstract similar to those used by Derry et al [3]. On further inspection of the results from Derry et al [3], 53% (53/100) of their MEDLINE records and 49% (43/88) of their EMBASE records contained an adverse effect indexing term compared to 80% (204/256) from DARE in our case study and 64% (9/14) from CDSR. This indicates that systematic reviews with a main outcome of an adverse effect may be marginally easier to retrieve than the RCTs in MEDLINE and EMBASE examined by Derry et al [3] and that this may be due in part to indexing. Indexers are instructed to index only the most important subject matter in an article [9], therefore, it may not be surprising that the RCTs in Derry et al's [3] study were not all indexed with adverse effects terms. It is surprising, however, that so many of the papers whose primary outcome was an adverse effect, such as those examined in this study, are not indexed with adverse effect terms.

The variation in reported sensitivities from the different case studies may reflect their different inclusion criteria. Derry et al [3] limited their analysis to RCTs whereas Badgett et al [2] and Golder et al [4] included all types of primary studies. RCTs in particular, may not have adverse effects as the main outcome of the study and, therefore, may not contain this information in the title or abstract. This in turn means there is less information for an indexer to identify and index.

Derry et al [3] and Badgett et al [2] did not measure precision. The precision of the search strategies reported in Golder et al [4] (0% to 9%) and the precision of the searches in CDSR in this study (0% to 3%) are similar. However, the precision of the searches in DARE are higher in this study. For example, searching with 'floating' subheadings gave a precision of 16% in this study compared with just 3% in Golder et al [4]. Searching for text words in the title and abstract gave 18% precision in this case study and 4% in Golder et al [4]. The largest discrepancy was seen when searching with the MeSH term Exp DRUG TOXICITY where 67% precision was achieved in this study (although with a very low sensitivity at 1%) compared to a precision of just 8% in Golder et al [4] (and sensitivity of 9%). This higher precision in DARE may reflect the relative size of DARE and MEDLINE. 5% (256/4919) of the total number of records on DARE were relevant to this case study and it would, therefore, not have been possible to achieve such low precision in DARE as that achieved by Golder et al [4] in MEDLINE.

### Limitations of the study

Our searches were limited to CDSR and DARE. Although these are excellent sources of systematic reviews of adverse effects, not all reviews reported as being systematic are contained in these databases: DARE has a strict quality inclusion criterion and CDSR contains only Cochrane Reviews. These databases are sources of systematic reviews that tend to be of higher methodological quality, which may reflect better reporting and hence better indexing.

The low number of systematic reviews of adverse effects on CDSR (14) precluded any useful analysis of the data, including comparisons to DARE and previous research. In addition, the usefulness of individual search terms was difficult to assess because of a low number of records.

The search terms tested in this study were predefined from previous research and were not obtained by objective methods [10]. However, the papers (n = 13) not retrieved by the searches used in this study did not reveal many additional relevant terms.

## Conclusion

Searching major systematic reviews databases for systematic reviews of adverse effects proved more difficult than anticipated due to a lack of standard terminology used by the authors of reviews, inadequate indexing and the variations in the search interfaces of these databases.

Our research suggests that it will be even more difficult to conduct thorough searches for systematic reviews that report adverse effects as a secondary outcome even in resources devoted to systematic reviews such as DARE and CDSR. At present hand searching all records in DARE and CDSR seems to be the only way to ensure retrieval of all systematic reviews of adverse effects in these databases.

## Key messages

Every systematic review with adverse effect(s) as a primary outcome should be indexed with appropriate term(s).

Authors of systematic reviews should use standardised terminology to make it explicit that they are reviewing adverse effects.

Database producers and indexers need to improve the consistency of their indexing of adverse effects.

The publishers of The Cochrane Library and the producers of DARE could increase the utility of these databases to users – the former by allowing searches to be limited to sections of the structured abstracts in both DARE and CDSR records, and the latter by introducing the facility to search DARE with 'floating' subheadings.

## Competing interests

The author(s) declare that they have no competing interests.

## Authors' contributions

SG participated in the conception and design of the study, carried out the searches, sifted the records for relevant reviews and carried out an evaluation of the searches and helped draft the manuscript. YL carried out sifting of records for relevant reviews and helped draft the manuscript. HM conceived the study, sifted the records for relevant reviews, and helped draft the manuscript. All authors read and approved the final manuscript.

## Pre-publication history

The pre-publication history for this paper can be accessed here:


